# Short-Term Delayed Recall of Auditory Verbal Learning Test Is Equivalent to Long-Term Delayed Recall for Identifying Amnestic Mild Cognitive Impairment

**DOI:** 10.1371/journal.pone.0051157

**Published:** 2012-12-07

**Authors:** Qianhua Zhao, Yingru Lv, Yan Zhou, Zhen Hong, Qihao Guo

**Affiliations:** 1 Department of Neurology and Institute of Neurology, Huashan Hospital, Fudan University, Shanghai, China; 2 Department of Radiology, Huashan Hospital, Fudan University, Shanghai, China; University of São Paulo, Brazil

## Abstract

Delayed recall of words in a verbal learning test is a sensitive measure for the diagnosis of amnestic mild cognitive impairment (aMCI) and early Alzheimer’s disease (AD). The relative validity of different retention intervals of delayed recall has not been well characterized. Using the Auditory Verbal Learning Test–Huashan version, we compared the differentiating value of short-term delayed recall (AVL-SR, that is, a 3- to 5-minute delay time) and long-term delayed recall (AVL-LR, that is, a 20-minute delay time) in distinguishing patients with aMCI (n = 897) and mild AD (n = 530) from the healthy elderly (n = 1215). In patients with aMCI, the correlation between AVL-SR and AVL-LR was very high (r = 0.94), and the difference between the two indicators was less than 0.5 points. There was no difference between AVL-SR and AVL-LR in the frequency of zero scores. In the receiver operating characteristic curves analysis, although the area under the curve (AUC) of AVL-SR and AVL-LR for diagnosing aMCI was significantly different, the cut-off scores of the two indicators were identical. In the subgroup of ages 80 to 89, the AUC of the two indicators showed no significant difference. Therefore, we concluded that AVL-SR could substitute for AVL-LR in identifying aMCI, especially for the oldest patients.

## Introduction

Episodic memory impairment is the core feature of Alzheimer’s disease (AD) and amnestic mild cognitive impairment (aMCI). Accurate testing for episodic memory deficits is an essential part of detecting early cognitive impairment. Basing on a word list, the verbal learning test (VLT) is a popular method of episodic memory detection; the VLT’s index of “delayed recall” is considered the most sensitive measure for the early diagnosis of AD. It is also regarded as the best predictor of conversion from MCI to AD [1].

A variety of standardized VLTs, such as the Rey Auditory Verbal Learning Test [2] (RAVLT, 1958), California Verbal Learning Test [3]–[4] (CVLT, Delis 1989; CVLT-II, 2000), and Hopkins Verbal Learning Test [5] (HVLT-R, Brandt and Benedict, 2001), are commonly used for clinical diagnosis and disease monitoring [6]. Other VLTs [7], used as a subtest for a battery of tests (for example, ADAS-cog [8] and CERAD [9], [10]), are easier and more convenient. These tasks present with relatively fewer words (a 10-word list) and fewer learning trials (2 to 3) (Table 1).

**Table 1:** Summary of the profile of commonly used verbal memory tests or tests that involve verbal memory.

Test name	Words list span	Learning trails	Retention interval for delay recall(min)		
			short delay	long delay	
**California verbal learning test (CVLT)**	16	5	1*		20
**Rey auditory verbal learning test (RAVLT)**	15	5	3–4#		20∼30
**Hopkins verbal learning test-revised (HVLT-R)**	12	3	/		20–25
**CERAD-CWL**	10	3	3–5		/
**the DemTect**	10	2	5–8		/
**Alzheimer’s disease assessment scale-cognition (ADAS-cog)**	10	3	5–8		/
**AB cognitive screen (ABCS)**	5	≤5	3–5		/
**Montreal cognitive assessment (MoCA)**	5	2	3–5		/
**Mini- mental state examination(MMSE)**	3	≤5	1–2		/
**Auditory verbal learning test-Huashan version (AVLT-H)**	12	3	3–5		20

CERAD-CWL: The Consortium to Establish a Registry for Alzheimer’s Disease 10-word list.

* after list B interference.

# after list B interference and a free-recall test of that list.

Besides the difference in lengths of word lists and numbers of trials, these verbal memory tasks also differ in testing procedure. The most obvious difference is the retention interval of delayed recall: standardized VLTs usually require 20 to 30 minutes (i.e., long-term delayed recall), whereas other VLTs require less than 10 minutes, mostly 2 to 5 minutes (that is, short-term delayed recall). As such, although word lists with delayed recall are well-established paradigms, the retention interval for delayed recall remains controversial.

The Auditory Verbal Learning Test–Huashan version (AVLT-H) [11] adopts the rationale and methods of the CVLT and Hong Kong Verbal Learning Tests. It includes short-term delayed recall (AVL-SR, that is, a 5-minute delay time) and long-term delayed recall (AVL-LR, that is, a 20-minute delay time). It is proved to be acceptable to Mandarin speakers and is sensitive to detecting aMCI. Many screening tests, such as the Mini-Mental State Examination (MMSE), the Montreal Cognitive Assessment (MoCA), and Dem Tect, use short-term delayed recall to detect memory impairment, and these tests have achieved acceptable validity. Therefore, we hypothesized that the diagnostic value of short-term (SR) and long-term delayed recall (LR) for aMCI is similar. Further, because age is a significant factor that correlates with memory decline, we also examine whether age affects the discriminating ability of the AVL-SR and AVL-LR for diagnosing aMCI.

## Methods

### Participants

The AD and aMCI patients were recruited consecutively at the Memory Clinic of Huashan Hospital from 2005 to 2010. Cognitively normal controls (NC), were enrolled using cluster sampling from Jingansi Community, Shanghai, China in a normal-aging study. The common inclusion criteria for all participants were as follows: (1) aged 50 to 89 years old; (2) formal education not less than 6 years; (3) adequate visual and auditory acuity to allow cognitive testing; (4) absence of significant medical or neurological diseases and psychiatric disorders or psychotic features that could compromise cognition. In total, the sample consisted of 1215 NC subjects, 897 aMCI patients, and 530 mild AD patients.

Criteria for aMCI [12], in addition to those outlined above, included the following: (1) memory complaints and memory decline, which were verified by an informant; (2) symptoms lasting more than 3 months; (3) total score on MMSE-Chinese version (C-MMSE) [13] (Katzman, 1988) not less than the education-adjusted cut-off scores; (4) abnormal objective memory impairment documented by the scores falling 1.5 SD below the age- and education-specific norms on one of the following two memory tests: the delayed recall of the Logical Memory Test (LMT, the paragraph recall test from the Wechsler Memory Scale-Chinese revised) and the delayed recall of the Rey-Osterreich Complex Figure Test (RCFT) [14]; (5) preserved basic activities of daily living and minimal impairment in complex instrumental functions, assessed on the basis of patient and informant interviews and ratings on a Functional Activities Questionnaire (FAQ) [15]; (6) etiology unknown; (7) non-demented according to the criteria of the National Institute of Neurological and Communicative Disorders and Stroke and the Alzheimer’s Disease and Related Disorders Association (NINCDS-ADRDA) [16].

The additional inclusion criteria for NC included the following: (1) cognitively normal, with no memory complaints or memory difficulties, verified by an informant; (2) global Clinical Dementia Rating (CDR) = 0 [17].

Mild AD was diagnosed according to the NINCDS-ADRDA criteria [16] and the global CDR = 1 [17].

To determine the age effect of delayed recall, participants were further divided into four subgroups: 50–59 years, 60–69 years, 70–79 years, and 80–89 years.

### Ethnic Issues

The study was approved by the Institutional Review Board of Huashan Hospital. Written informed consent was obtained from all the participants.

### Procedures

Each subject had a uniform structured evaluation performed by a neurologist, which included a medical history inquiry and neurological examination. Blood tests included complete blood count, thyroid function tests, serum vitamin B_12_, and Venereal Disease Research Laboratories test. CT or MRI scans were performed for all the participants. A comprehensive neuropsychological battery including memory, language, attention, executive functioning, and visuospatial ability was administered. The tests were as follows: the C-MMSE [13], the LMT [18], the RCFT [19], the Boston Naming Test (the 30-item version) [20], [21], the Animal Verbal Fluency Test [22], the Symbol Digit Modalities Test [23], the Trail Making Test–A and B [24], the Stroop Color-Word Test [25], the Similarity Test [26], the Clock-drawing Test [27], the CDR [17], and the FAQ [15]. All these tests have been proved to have good reliability and validity in Chinese. The neuropsychological tests were performed by three highly trained raters (Y Zhou, YM Sun, and MR Chen). The diagnoses were kept blind to the raters.

**Table 2:** Demographic characteristics and AVLT-H indicators in age-specific diagnostic groups.

Index		50–59 yrs				60–69 yrs				70–79 yrs				80–89 yrs		
	NC	aMCI	AD	F(P)	NC	aMCI	AD	F(P)	NC	aMCI	AD	F(P)	NC	aMCI	AD	F(P)
**N**	420	222	136		376	255	111		316	325	199		103	95	84	
**Sex (M:F)**	181:239	100:122	63:73	0.25 (0.77)	173:202	138:117	50:61	2.29 (0.10)	175:141	187:138	114:85	0.17 (0.84)	65:38	51:44	43:41	1.54 (0.21)
**Age**	55.47 (3.33)	54.98(3.41)	55.10 (55.26)	1.82 (0.16)	65.06 (2.50)	65.32 (2.60)	65.18 (2.64)	0.83 (0.43)	73.72 (2.63)	74.06 (2.82)	74.14 (2.69)	1.86 (0.15)	83.84 (2.90)	83.75 (2.61)	83.88 (3.08)	0.04 (0.95)
**Education**	10.55 (2.75)	10.74(2.64)	10.23 (2.73)	1.45 (0.23)	12.16 (2.98)	11.87 (2.98)	11.57 (2.81)	1.89 (0.15)	11.68 (3.27)	11.66 (3.22)	11.17 (3.30)	1.75 (0.17)	12.25 (3.20)	12.18 (3.67)	11.53 (3.27)	1.21 (0.29)
**MMSE**	28.38 (2.69)	27.22 (1.98)**	19.97 (3.88) †† ^##^	482.33 (0.00)	28.21 (2.86)	26.78 (1.97)**	20.49 (3.44) †† ^##^	352.39 (0.00)	27.89 (1.75)	26.65 (1.86)**	20.19 (3.43) †† ^##^	743.15 (0.00)	27.57 (1.88)	26.35 (1.90)**	20.73 (2.68) †† ^##^	254.94 (0.00)
**AVL-T**	17.87 (4.69)	12.08 (3.82)**	7.92 (3.95) †† ^##^	316.40 (0.00)	17.69 (4.83)	11.36 (3.73)**	7.57 (3.71) †† ^##^	305.66 (0.00)	16.18 (4.42)	10.81 (3.33)**	7.19 (3.42) †† ^##^	364.95 (0.00)	14.45 (3.70)	9.41 (3.13)**	7.03 (3.40) †† ^##^	116.02 (0.00)
**AVL-SR**	6.57 (2.05)	2.40 (2.03)**	0.55 (1.29) †† ^##^	647.40 (0.00)	6.16 (2.17)	1.78 (1.77)**	0.35 (1.10) †† ^##^	605.18 (0.00)	5.56 (1.97)	1.39 (1.65)**	0.38 (0.94) †† ^##^	770.80 (0.00)	5.01 (1.77)	1.22 (1.42)**	0.36 (0.95) †† ^##^	281.11 (0.00)
**AVL-LR**	6.44 (1.89)	2.09 (1.84)**	0.48 (1.20) †† ^##^	784.73 (0.00)	6.00 (2.19)	1.39 (1.57)**	0.24 (0.89) †† ^##^	679.79 (0.00)	5.24 (1.96)	1.04 (1.32)**	0.28 (0.87) †† ^##^	872.21 (0.00)	4.80 (1.91)	0.81 (1.16)**	0.20 (0.67) †† ^##^	314.76 (0.00)

Comparison between NC group and aMCI group was marked behind ‘aMCI group’;

** P<0.01.

Comparison between aMCI group and AD group was marked behind ‘AD group’;

† P<0.05;

†† P<0.01.

Comparison between NC group and AD group was marked behind ‘AD group’. P<0.05;

## P<0.01.

### AVLT-H

The word list is composed of 12 two-character words from three semantic categories (flowers, occupations, apparels) with four words for each category. The AVLT-H measures both recall and recognition of the word lists over a number of trials. Administration of the test begins by evaluating an individual’s ability to recall the 12-word list over three learning trials. A non-verbal test (copy part of RCFT) is then presented for interference with a 3–5-minute interval. The Short-term Delayed Free Recall follows. After a 20-minute delay during which a nonverbal task (including the Symbol Digit Modalities Test and the Trail Making Test) occurs, free recall is again tested (that is, Long-term Delayed Free Recall), as well as category-cued recall. Finally, a recognition test that includes the 12 target words and 12 distracter words (semantically related) is performed.

The test scores of the AVLT-H are as follows: (1) AVL-T, sum of all correct responses given in the first three consecutive trials; (2) AVL-SR, number of words responded correctly in the short-term delayed free recall; (3) AVL-LR, number of words responded correctly in the long-term delayed free recall; (4) AVL-CR, number of words answered correctly with category-cued recall; (5) AVL-REC, number of words answered correctly in the recognition test. Because we sought to explore the recession of episodic memory function, our target variables excluded the AVL-T, AVL-CR, and AVL-REC.

**Figure 1:**
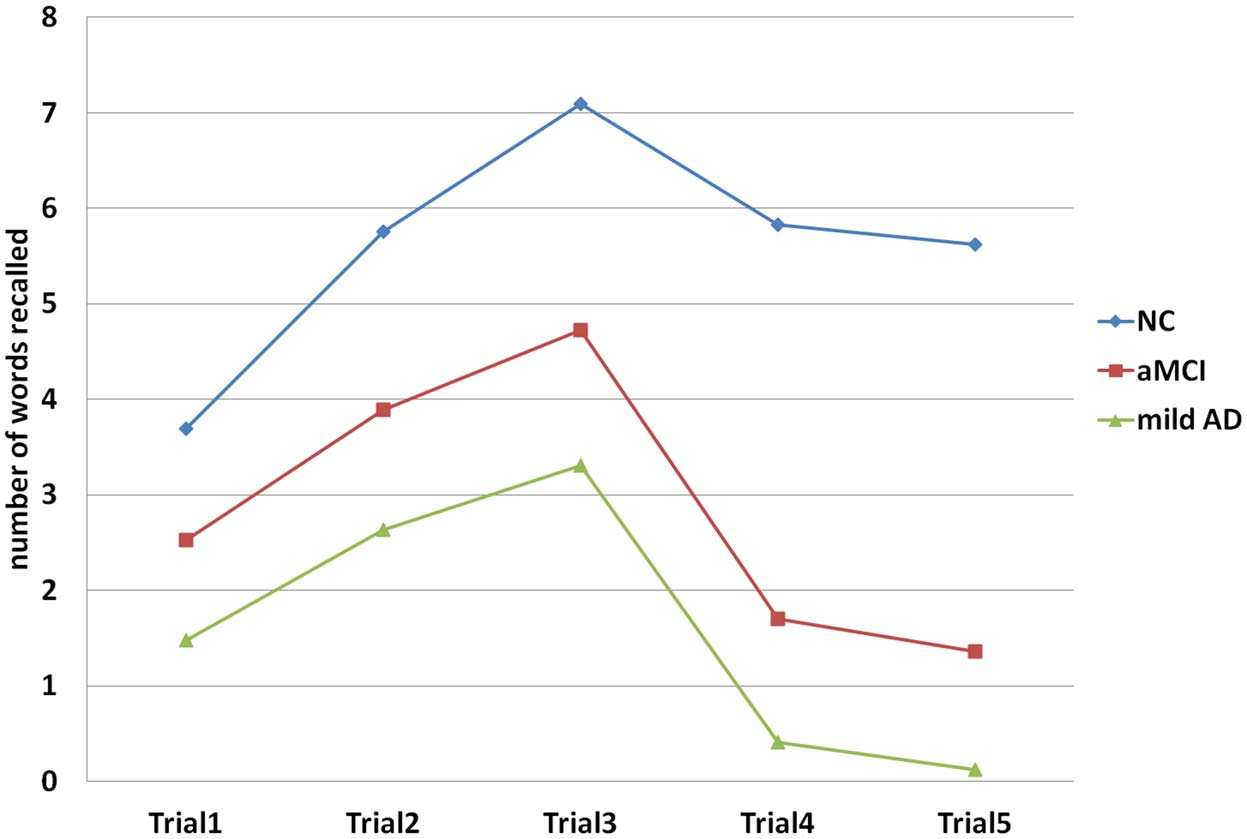
AVL scores in three diagnostic groups in each trial.

**Figure 2:**
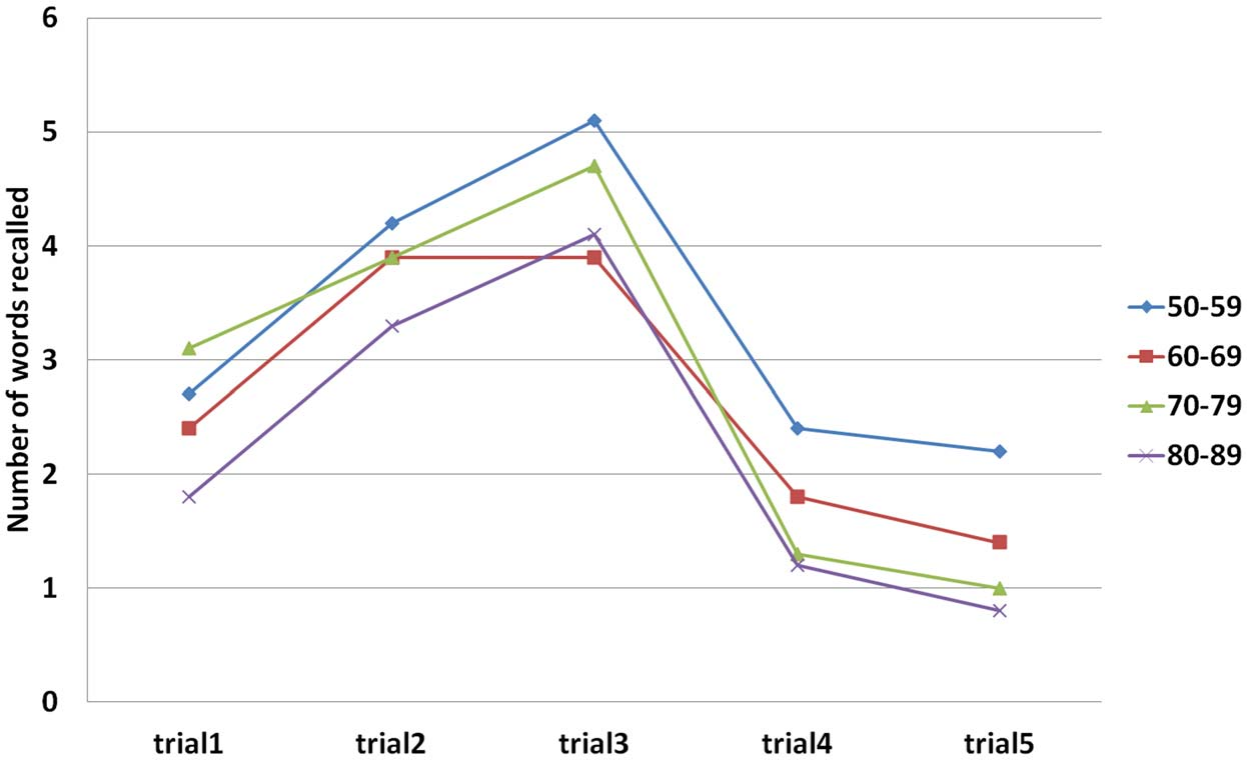
AVL scores of different age groups in each trial in patients with aMCI.

### Statistical Analysis

Chi-square analysis was adopted for ordinal data. Overall continuous variables among the three groups (aMCI, mild AD, and NC groups) were assessed with one-way analysis of variance. Post hoc pairwise comparisons between groups were assessed using the LSD test. Receiver operating characteristic (ROC) curves were used to determine the ability of AVL-SR and AVL-LR to discriminate aMCI from NC. The level of significance was set at α = 0.05. Statistical analyses were carried out using SPSS 16.0.

**Table 3:** Percentage of zero scores of the AVL-SR and AVL-LR in different age groups in patients with aMCI.

	Percentage of zero score of AVL-SR	Percentage of zero score of AVL-LR	?^2^	P value
**50–59 (n = 222)**	27.5%	29.3%	0.177	0.674
**60–69 (n = 255)**	39.2%	45.1%	1.809	0.179
**70–79 (n = 325)**	48.0%	52.3%	1.206	0.272
**80–89 (n = 95)**	48.4%	58.9%	2.117	0.146

## Results

### Demographic Characteristics of Three Diagnostic Groups

Table 2 summarizes demographic information, MMSE, and AVLT-H scores for the three diagnostic groups (NC, aMCI, mild AD). Patients in the three groups were comparable in age, education level, and gender distribution.

**Table 4:** ROC analysis of AVL-SR and AVL-SR for identifying aMCI in different age groups.

	Index	ROC AUC(95% CI)	Cut-off	Sensitivity (%)	Specificity (%)
**50–59years**	AVL-SR	0.919 (0.896∼0.942)	≤4	84.9	84.3
	AVL-LR	0.952 (0.934∼0.970)	≤4	87.4	92.6
**60∼69years**	AVL-SR	0.944 (0.927∼0.961)	≤3	92.6	80.0
	AVL-LR	0.961 (0.947∼0.976)	≤3	92.6	91.4
**70∼79years**	AVL-SR	0.943 (0.926∼0.960)	≤2	96.6	72.7
	AVL-LR	0.962 (0.949∼0.975)	≤2	95.6	85.2
**80–89years**	AVL-SR	0.948 (0.921∼0.976)	≤2	94.2	76.8
	AVL-LR	0.964 (0.942∼0.986)	≤2	94.2	89.5

ROC: receiver operating characteristic curve.

AUC: area under the curve.

95% CI: 95% confidence interval.

### Correlations between Demographic Variables and AVLT-H Indicators

Age was negatively correlated with AVL-SR and AVL-LR (r = −0.24 and −0.26, p<0.01) in NC group. Both indicators had a significant gender difference (p<0.01). The higher difference was found in AVL-LR with an average score of 5.3 for males and 6.3 for women. Scores on the AVLT indicators were not significantly related to education (r = 0.01–0.03, p> 0.05).

### Correlations between AVL-SR and AVL-LR

For all participants, the correlation between AVL-SR and AVL-LR was very high (r = 0.94, p<0.01). Regardless of cognitive status and age, the difference in mean value between AVL-SR and AVL-LR was very small, less than 0.5 points, and not significant (p>0.5). For delayed recall, from 5 minutes to 20 minutes, the score curve was shaped like a platform (see Figures 1 and 2).

### Percentage of Zero Scores of AVLT-H Indicators

A score of zero for AVLT-H also reflects memory impairment in patients. Thus we compared the percentage of zero scores on AVL-SR and AVL-LR. No significant difference was found between the two indicators in aMCI (Table 3).

### ROC Analysis of AVLT Indicators for Identifying aMCI in Different Age Groups

In the 80- to 89-year-old group, ROC areas under the curve (AUCs) were similar between AVL-SR and AVL-LR (Z = 1.76, p = 0.08). In other age groups (that is, the 50–59, 60–69, and 70–79 age groups), the AUC of AVL-LR was greater than that for AVL-SR (Z = 4.28, 2.93, 2.97, respectively, p<0.01) (Table 4).

## Discussion

To our knowledge, there is little information detailing the diagnostic utility of different retention intervals of delayed recall for aMCI, the prodromal stage of AD. The present study used a case-control design to determine the diagnostic value of using short-term and long-term delayed recall performance of the AVLT-H for aMCI identification. We also measured age-specific classification accuracy for the two delayed recall indicators.

Long-term delayed recall of word list learning appeared to have the highest diagnostic accuracy for differentiating MCI patients from controls and might provide the most specific measure for early AD diagnosis. Long-term delayed recall reflects entorhinal and hippocampal cortical function, where the earliest neuropathological changes in AD occur [28], [29]. Retention intervals of delayed recall have to be long enough to be sensitive to impairment but short enough to keep the examinee’s compliance. So far, few have compared the use of AVL-SR and AVL-LR in identifying aMCI. In the current study, both indicators had ideal diagnostic value. Moreover, in the 80- to 89-year-old group, the AUC of AVL-SR and AVL-LR showed no significant difference.

The AVLT-H has been shown to accurately distinguish between aMCI and controls. Our study shows that the correlation between AVL-SR and AVL-LR was very high (r = 0.94), and the percentage of zero scores for the two indicators was similar. The numerical difference of the two indicators was less than 0.5 points in each age group of aMCI. There were few studies focusing on the time interval of delayed recall in VTLs. The Alzheimer’s Disease Neuroimaging Initiative (ADNI) group once examined the Rey AVLT measures and brain volume. They reported that, for both 5-minute and 30-minute delayed recall trials, the hippocampus was the only region correlated with the performance [28]. The ADNI results suggested a common biological basis of short-term and long-term delayed recall. They did not, however, provide direct evidence of the equivalence between the two trials. It is worth noting that, in the current study, although the AUCs for the two indicators were different in some age groups, the cut-off scores were identical. Practically, the AVL-SR appeared to be sensitive enough to detect a memory deficit. It was the first time that the close correlation of AVL-SR and LR had been reported in a Chinese version of verbal learning test.

To identify MCI and determine its subtypes, it is necessary to assess multiple cognitive domains such as memory, language, attention, and visual-spatial and executive function. A comprehensive neuropsychological test battery is always time-consuming, especially the memory test. There is an urgent need for a test that requires minimal time investment but maintains the clinical diagnosing value for subtle memory decline like in MCI. But if the content and procedure of a classical test is arbitrarily reduced without re-validation, the accuracy and reliability of the test will undoubtedly be affected. The current study suggests that, using a relatively shorter time interval (like AVL-SR), it is possible to identify aMCI in a feasible time duration, especially for the oldest (80–89 years old) with poor physical and psychological tolerance. Yet, if time permits, the full version of the VLT including AVL-LR is still recommended for a more accurate and sensitive measurement.

The present study has some strength. Three memory tests including LMT, RCFT, and AVLT were administered to all the participants. The aMCI was diagnosed according to the scores of long-term delayed recall of both LMT and RCFT, whereas the AVLT-H scores were not taken into consideration for diagnosis, thus avoiding a circular argument. Additionally, the relatively large sample size allowed the analysis to be performed in age-stratified groups, which minimized the confounding effect of age. There was, however, an important limitation as well. Without AD-specific biomarker analysis such as beta-amyloid and Tau protein measurement, we could only base our diagnosis of AD and MCI on clinical evidence in a probable level of likelihood [30], [31].

In general, short-term delayed recall (that is, 3- to 5-minute delay time) and long-term delayed recall (that is, 20-minute delay time) of AVLT-H has a similar ability to discriminate aMCI from cognitively normal subjects. Future research will assess the value of each AVLT-H score in the differential diagnosis, prognosis, and conversion prediction of MCI to dementia.
